# Chest pain score: a novel and practical approach to angina pectoris. A diagnostic accuracy study

**DOI:** 10.1590/1516-3180.2018.0238101218

**Published:** 2019-05-08

**Authors:** Fatih Aydin, Ercan Aksit, Ozge Turgay Yildirim, Ayse Huseyinoglu Aydin, Evrin Dagtekin, Murat Samsa

**Affiliations:** I MD. Physician, Department of Cardiology, Eskisehir State Hospital, Eskisehir, Turkey.; II MD. Physician, Department of Cardiology, Canakkale Onsekizmart University, Canakkale, Turkey.; III MD. Physician, Department of Cardiology, Eskisehir State Hospital, Eskisehir, Turkey.; IV MD. Physician, Department of Cardiology, Eskisehir State Hospital, Eskisehir, Turkey.; V MD. Physician, Department of Cardiology, Eskisehir State Hospital, Eskisehir, Turkey.; VI MD. Physician, Department of Cardiology, Selcuk State Hospital, Izmir, Turkey.

**Keywords:** Chest pain, Angina pectoris, Myocardial ischemia

## Abstract

**BACKGROUND:**

The chest pain classifications that are currently in use are based on studies that are several decades old. Various studies have indicated that these classifications are not sufficient for determining the origin of chest pain without additional diagnostic tests or tools. We describe a new chest pain scoring system that examines the relationship between chest pain and ischemic heart disease (IHD).

**DESIGN AND SETTING:**

Cross-sectional study conducted in a tertiary-level university hospital and two public hospitals.

**METHODS:**

Chest pain scores were assigned to 484 patients. These patients then underwent a treadmill stress test, followed by myocardial perfusion scintigraphy if necessary. Coronary angiography was then carried out on the patients whose tests had been interpreted as positive for ischemia. Afterwards, the relationship between myocardial ischemia and the test score results was investigated.

**RESULTS:**

The median chest pain score was 2 (range: 0-7) among the patients without IHD and 6 (1-8) among those with IHD. The median score of patients with IHD was significantly higher than that of patients without IHD (P = 0.001). Receiver operating characteristic analysis showed that the score had sensitivity of 97% and specificity of 87.5% for detecting IHD.

**CONCLUSION:**

We developed a pre-test chest pain score that uses a digital scoring system to assess whether or not the pain was caused by IHD. This scoring system can be applied easily and swiftly by healthcare professionals and can prevent the confusion that is caused by other classification and scoring systems.

## INTRODUCTION

Chest pain is the most common complaint leading to hospital admission^1^^,^^2^ and is a common symptom of numerous diseases.^3^ All physicians, and particularly cardiologists, want to rule out ischemic heart disease (IHD) as fast as possible when they are confronted with a patient complaining of chest pain. The patient’s history is crucial for the diagnosis of IHD; therefore, history-taking needs to be done with the utmost care.^4^

Currently, the chest pain classification defined by Diamond in the 1980s is still used (with minor changes) to determine whether or not the cause of the chest pain is myocardial ischemia.^5^ Three features of the pain are evaluated in this classification: (1) the duration of the chest pain (which can range from 2 to 15 minutes) and degree of discomfort, and whether it is located in the retrosternal region; (2) whether the discomfort was triggered by effort or emotional stress; and (3) whether the pain promptly disappeared after rest and/or nitrate administration. If the characteristics of the chest pain include all three features mentioned above, the pain is classified as “typical chest pain”. If only two of these features are present, the pain is classified as “atypical chest pain”. If only one or none of these features is present, the pain is classified as “non-cardiac chest pain”.

However, this classification is not sufficient for making the diagnosis of ischemia in the absence of stress tests.^6^^,^^7^ Furthermore, these statements may result in erroneous results among elderly patients and those with diabetes mellitus, because pain perception may be impaired in these groups.^8^^,^^9^ Anotherproblem is that this classification is largely subjective, which results in disagreements among physicians and even among cardiologists, as to what constitutes “typical/atypical” chest pain.

Symptoms that may be typical for IHD can be overlooked in cases with prolonged resting angina or angina that was triggered by an effort so minimal that the patients did not realize that the symptom was caused by this effort. Furthermore, lung diseases or peripheral arterial diseases may restrict exercise capacity and the complaints of these patients may very well subside with rest. These disorders may cause increases in symptoms of chest pain during exercise and decreases in discomfort during resting period, thus causing high suspicion of angina pectoris, even though the origin of the symptoms was non-cardiac.

In addition to these problems, as mentioned in several studies and as seen in our own clinical experience, the nomenclature of “typical”, “atypical” and “non-cardiac” is often insufficient and may lead to misunderstandings.^6^ Therefore, it is apparent that a new evaluation method that can accurately identify cardiac chest pain without any such pitfalls is required.

## OBJECTIVE

In this study, our aim was to create a chest pain score that was accurate, swift, more comprehensive and more objective in determining whether or not the chest pain in question was related to IHD.

## METHODS

### Study group

All patients who presented to the cardiology clinics or emergency departments (ER) of Eskisehir State Hospital (a secondary-level healthcare center) or Canakkale Onsekiz Mart University Hospital (a tertiary-level healthcare center) complaining of chest pain between June 2017 and February 2018 were included in this study. Patients with prior ST segment elevation myocardial infarction, pregnant patients and individuals under 18 years of age were excluded from the study.

After exclusion of any patients who refused to participate in the study and those presenting exclusion criteria, data were collected from the remaining subjects, as our sample of patients. Acardiology specialist applied the chest pain score to all subjects through face-to-face interviews.

### Ethical approval

Implementation of this study was endorsed by our institution’s Internal Review Board (date: September 27, 2017; number:2017-15).

### Risk score development

After the patients had been assessed for typical risk factors such as age, gender, diabetes mellitus, hypertension, coronary artery diseases (CAD) and smoking status, the chest pain score developed for this study was applied to them.

Data collection was planned before the index test was performed and before the reference standard was implemented. Agroup consisting of 10 experienced cardiology specialists was given the task of creating the scoring system. This group initially determined which cardiac complaints were typical, according to their own clinical experience, and this was followed by meticulous evaluation of the literature on this topic.

A total of 10 major questions regarding chest pain properties and the patients’ characteristics were formulated and evaluated by this group of 10 cardiologists. Through this assessment, unnecessary questions were omitted, such that the number of questions was reduced to five. The test consisting of these five questions was then applied to a preliminary group of 20 patients and the questions were then weighted according to the results from this test.

The final chest pain scoring system that was proposed in the present study thus consisted of five questions that could only be answered as “yes” or “no”. “No” answers were scored as zero points. For the first and fifth questions, a “yes” answer was scored as 2 points. For the second and fourth questions, a “yes” answer was scored as 1point. The third question consisted of two sub-questions: for each sub-question, a “yes” answer was counted as 1 point, such that the maximum score was 2 points. After the total score from the questions had been obtained, 1 point was added to the score of patients with diabetes and/or those older than 75 years of age (Table 1).

**Table 1.** Chest pain score

Chest pain:	No	Yes
1. Is it in the form of pressure, fullness, burning, discomfort or tightness in your chest?	0	2
2. Is the duration of chest pain less than 10-15 minutes but longer than about a minute?	0	1
3. a) Is it behind the sternum? (spreading on the sternum, not localized) b) Is it in the left or right arm in the ulnar part, the lower cannula, the epigastric region, the scapula region, or does it radiate to these regions?	0 0	1 1
4. Is it accompanied by shortness of breath, sweating, nausea, fatigue or syncope?	0	1
5. Is it triggered by effort or emotional stress and eased by rest or nitrates?	0	2

One point is added to the sum if diabetes mellitus is present and/or the patient is > 75 years old.

After final scores for the patients evaluated in this study had been obtained, patients who had been found to present a high risk of ischemic heart disease underwent coronary angiography without a stress test. The remaining patients then underwent a treadmill stress test. The treadmill stress test was considered positive for patients who presented 1 mm of horizontal or downsloping ST segment depression in three consecutive leads, or ST segment elevation, or chest pain triggered by the stress test. Myocardial perfusion scintigraphy (MPS) was performed on the patients with suspicious treadmill stress test results. MPS imaging was performed using the imaging protocol developed by the American Society of Nuclear Cardiology.^10^ Lastly, coronary angiography was performed on patients who had positive non-invasive ischemia test results.

The patients were divided into three groups depending on the results from coronary angiography. Patients with > 50% stenosis in the left main coronary artery (LMCA) or > 70% stenosis in any vessel with a diameter > 2 mm were classified as the “critical coronary artery disease” group. Patients with any lesion not classified as critical or patients with slow coronary flow were classified as the “non-critical coronary artery disease” group. Patients with normal coronary arteries were classified as the “normal coronary arteries” group. The ischemia-positive group included the critical CAD patient group, the non-critical CAD patient group and the group of patients whose non-invasive stress tests were positive. Patients with a positive stress test and normal coronary arteries were included in the ischemia-negative group.

### Statistical analysis

The IBM Statistical Package for the Social Sciences (SPSS) software for Windows, version 15.0 (IBM Corp. Armonk, NY, USA), was used for data analysis. The demographic characteristics of the study group were reported using descriptive statistics (frequencies, proportions, means and medians) and dispersion measurements (standard deviation and minimum-maximum). Initially, the normality of the total scores was tested using the Kolmogorov-Smirnov normality test and graphs. Frequency data were analyzed by using the chi-square test as univariate analysis. Receiver operating characteristic (ROC) analysis was used for calculating the sensitivity, specificity and positive and negative predictive values of cutoff scores from the scale. Median scores were compared using the Mann-Whitney U and Kruskal-Wallis tests. If any comparison yielded a P value less than 0.05, it was considered statistically significant.

## RESULTS

A total of 484 patients were included in this study. The patients’ mean age (± standard deviation, SD) was 52.0 ± 15.0 years (minimum-maximum: 18-84) for the entire study group, while the mean for males was 52.0 ± 14.0 years and the mean for females was 52.0 ± 15.0 years (P = 0.585). The study group consisted of 229 males (47.3%) and 255 females (52.7%).

The median chest pain score of those with positive treadmill stress test results was found to be 6, while the median score of the patients with negative results was 1. The scores of those who had positive exercise test results were significantly higher than the scores of those who had negative results (P < 0.001).

Regarding MPS results, those with positive results had a median score of 6, while those with negative results had a median score of 3. The chest pain scores of MPS-positive patients were significantly higher than the scores of MPS-negative patients (P < 0.001).

Comparison of the chest pain scores of patients with normal carotid arteries and those with critical and non-critical CAD showed that the median score of the critical and non-critical CAD group (median score: 6) was significantly higher than the score of those with normal carotid arteries (median score: 3) (P < 0.001).

The median chest pain scores for hypertensive and non-hypertensive patients were 3 and 2, respectively; while the scores for diabetic and non-diabetic patients were also found to be 3 and 2 respectively. Statistical analysis revealed that the scores for hypertensive and diabetic patients were significantly higher than the scores for those without the respective conditions (P < 0.001). Furthermore, it was observed that the chest pain score increased with age and that there was a significant relationship between age and chest pain score (P = 0.001). Regarding sex, the median chest pain scores were 3 and 2 for males and females, respectively. Thechest pain scores of males were significantly higher than those of females. There was no significant relationship between chest pain score and the history of CAD, smoking status, peripheral artery disease or heart failure. The chest pain score was 6 for IHD patientsand 2 for non-IHD patients. The chest pain score for IHD patients was significantly higher (P = 0.001). The relationships between ischemia and chest pain score are shown in Table 2.

**Table 2.** Relationship between ischemia and chest pain score

Questions	Ischemia-negative n (%)	Ischemia-positive n (%)	x^2^; P
Question 1			
0 points	278 (57.4%)	10 (2.1%)	128,186
2 points	106 (21.9%)	90 (18.6%)	< 0.001
Question 2			
0 points	203 (41.9%)	29 (6.0%)	18,105
1 point	181 (37.4%)	71 (14.7%)	< 0.001
Question 3			
0 points	231 (47.7%)	9 (1.9%)	
1 point	132 (27.3%)	44 (9.1%)	141,296
2 points	21 (4.3%)	47 (9.7%)	< 0.001
Question 4			
0 points	322 (66.5%)	73 (15.1%)	6,228
1 point	62 (12.8%)	27 (5.6%)	0.013
Question 5			
0 points	360 (74.4%)	30 (6.2%)	206,037
1 point	24 (5.0%)	70 (14.5%)	< 0.001

The patients were divided into four groups according to chest pain score, presence of IHD and pre-test risk factors for IHD (Table3). The first group was defined as “low IHD risk”, consisting of patients with scores of 0-2. There were two IHD patients and 255 non-IHD patients in the first group (2:255). The second group consisted of patients with a score of 3-4 and was defined as the “moderate IHD risk” group. There were eight patients with IHD and 113 without IHD in this group (8:113). The third group included patients with a score of 5-6 and was defined as the “high IHD risk” group. The ratio of patients with IHD (n = 57) to those without IHD (n = 15) in this group was 57:15. The fourth group consisted of patients with a score of 7-8 and was defined as the “very high IHD risk” group. The ratio of patients with IHD (n = 33) to those without IHD (n = 1) in this group was 33:1. There were significant differences between the groups in terms of IHD (P < 0.001).

**Table 3.** Risk groups and relationships with ischemia

Score points	Ischemia-negative n (%)	Ischemia-positive n (%)	x^2^; P
0-2 points (Low risk)	255 (52.7%)	2 (0.4%)	347,954 < 0.001
3-4 points (Moderate risk)	113 (23.3%)	8 (1.7%)	
5-6 points (High risk)	15 (3.1%)	57 (11.8%)	
7-8 point (Very high risk)	1 (0.2%)	33 (6.8%)	

**Figure 1.**
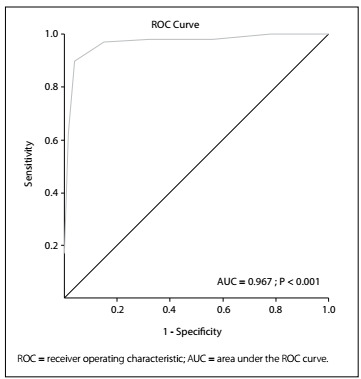
ROC curve of total score (cutoff: 4.5).

We also performed ROC curve analysis for each of the questions and for the total score. The results showed that a chest pain score threshold of 4.5 demonstrated sensitivity of 90% and specificity of 95.83% for detecting IHD, while the positive predictive value (PPV) and negative predictive value (NPV) were found to be 84.91% and 97.35%, respectively (Figure 1). The ROC analysis also revealed that question 4 could not differentiate between patients with and without IHD; however, when the analysis was repeated without question 4, significantly lower specificity and PPV were observed. Therefore,although the question itself could be considered unsuccessful, it was effective in the overall results and was not omitted (Table 4).

**Table 4.** ROC curve analysis results

	Sensitivity	Specificity	PPV	NPV	Area under the ROC curve				P	Cutoff
					Area	Standard error	Confidence interval			
Total score	90.00	95.83	84.91	97.35	0.967	0.011	0.946	0.988	< 0.001	4.5
Question 1	90.00	72.40	45.92	96.53	0.812	0.023	0.768	0.856	< 0.001	1.0
Question 2	71.00	52.86	28.17	87.50	0.619	0.031	0.559	0.679	< 0.001	0.5
Question 3	91.00	60.16	37.30	96.25	0.825	0.023	0.779	0.870	< 0.001	0.5
Question 4	27.00	83.85	30.34	81.52	0.554	0.033	0.489	0.620	0.094	0.5
Question 5	70.00	93.75	74.47	92.31	0.819	0.028	0.763	0.875	< 0.001	1.0
Total score (without Q4)	97.00	87.50	66.90	99.12	0.964	0.012	0.941	0.987	< 0.001	3.5

ROC = receiver operating characteristic; PPV = positive predictive value; NPV = negative predictive value.

## DISCUSSION

In this study, we applied a chest pain scoring system consisting of five questions, to patients who had complaints of chest pain, to determine whether or not the chest pain originated from IHD. The study was initially planned because we noticed that the classical classifications for chest pain (typical, atypical and non-cardiac) were insufficient without additional stress tests.^6^^,^^7^ Furthermore, Luke et al. reported that typical angina patients may not have inducible myocardial ischemia, while myocardial ischemia may be induced in patients with atypical angina, which means that the current nomenclature can lead to serious errors.^6^ Therefore, we aimed to create a better alternative to the current chest pain score system.

The classical chest pain classification created by Diamond suggests that two positive answers among the three questions that evaluate the association between exercise and pain (except the first question) are enough to determine that the source of pain is myocardial injury. However, in cases of acute coronary syndrome, pain may be triggered without stress or exertion.^11^^,^^12^^,^^13^ A study of the literature concerning stable angina pectoris revealed that vasospastic angina (a type of resting angina) is considered to be stable angina.^14^^,^^15^ However, this pain type is considered non-cardiac according to the aforementioned classical chest pain classification.

Another widely accepted chest pain classification score, the WHO-Rose angina questionnaire,^16^ is similar to the Diamond classification in terms of pain evaluation. This questionnaire was first introduced by Rose et al. in the 1970s and was later adopted by the World Health Organization (WHO), hence the name. In the following years, the WHO-Rose angina questionnaire was shortened and the final form comprised three questions.^16^ However, this questionnaire also overlooks cases in which patients may misinterpret their pain due to factors such as age and diabetes.

Given these facts, we aimed to develop a chest pain scoring system that would inherently solve this problem and could be implemented by all physicians regardless of specialty, and also by other medical staff. The proposed chest pain score is a swift and easy method for determining whether the chest pain in question is caused by myocardial injury, and it can be used in both the ER and the outpatient setting. The scoring system in this study can be used to assess patients’ risk of ischemia and can group patients based on these scores.

In this scoring system, we tried to evaluate the association between patients’ complaints and the presence of ischemia, without initially considering risk factors for cardiovascular diseases. In establishing this score, we took into account the four features of chest pain, as described in the European Cardiology Community guidelines: the characteristics of the pain, its location and extent, its duration and its relationship with exercise; while also evaluating other symptoms that accompany chest pain.^17^

In the past, various scoring systems for evaluating chest pain were proposed.^18^ However, the majority of these scoring systems included risk factors, electrocardiography findings and laboratory results (such as troponin levels).

The present chest pain assessment score is the first, since publication of the chest pain classification of 1983,^5^ to classify pain and examine its relationship with ischemia via questions that focus only on chest pain and its characteristics. We used this score to calculate the likelihood of pre-test ischemia from symptoms alone and to determine numerical values indicating the necessity for non-invasive tests. We also tried to define standard expressions that would be easier to understand in all healthcare environments, instead of the current “typical, atypical and non-cardiac” classifications, through examining the likelihood of ischemia using four separate risk groups categorized as low, medium, high and very high. Theresults from our analysis demonstrated that the proposed chest pain score increases with increasing likelihood of ischemic heart disease.

Old age and diabetes are risk factors for IHD but are also misleading factors for physicians because they can affect the anamnesis of patients. Since chest pain and insufficient capacity for exertion can be explained by diabetes and old age, their association with ischemia may be overlooked. Experiencing pain during exertion is a very specific finding in IHD, but this is difficult to detect in elderly patients because they are often not active enough to sense effort-related chest pain. It has been reported in many studies on diabetes and the elderly that pain can be expressed atypically even when it is associated with IHD.^8^^,^^9^^,^^19^ However, the classical chest pain classification and the WHO-Rose questionnaire do not take these characteristics into account, and therefore may cause misdiagnosis. Through the chest pain score in this study, we believe that physicians’ ability to identify ischemia in such patients is increased, even though we do not evaluate risk factors in the scoring system. This particular feature is achieved through accurate evaluation of atypical pain in patients whose cases could be overlooked if the classical chest pain questionnaires are used, because of differences in pain perception among patients.

The ROC analysis on the scoring system showed that this system has very high sensitivity and specificity for detecting IHD. Although question 4 was not successful on its own, for discriminating whether pain was associated with IHD, it was effective within the overall sensitivity and specificity of the scoring system. Therefore, the question was not omitted.

Although men were found to have significantly higher chest pain scores in the current study, we did not attempt to change our scoring system to evaluate differences between the sexes. This was because various studies have shown that there is no significant correlation between sex and the explanations for typical and atypical pain in patients with angina.^20^

This study was conducted with 484 patients in two separate centers. This may be considered to be a limitation of the study. Repeating this work with larger populations and different ethnic groups might yield different results. In this study, we used coronary angiography in an attempt to exclude false positive results from non-invasive tests, but the results may still have been impaired by false negative tests. However, we were unable to perform coronary angiography to exclude false negative results due to ethical concerns. There is a need to support this work through using larger populations and multinational studies in which patients are followed for longer periods.

## CONCLUSION

We developed a chest pain score that can easily and rapidly be applied by all healthcare workers, and which focuses solely on patients’ chest pain characteristics. The results from ROC analysis indicate that the proposed chest pain scoring system is very successful in identifying patients with pain due to cardiac injury. We believe that this scoring system can be used safely to accurately identify IHD in patients who present chest pain, without the use of non-invasive stress tests.
